# Enhanced bonding strength between lithium disilicate ceramics and resin cement by multiple surface treatments after thermal cycling

**DOI:** 10.1371/journal.pone.0220466

**Published:** 2019-07-25

**Authors:** Rui Li, Shi Qing Ma, Cheng Cheng Zang, Wen Yi Zhang, Zi Hao Liu, Ying Chun Sun, Yi Yu Feng

**Affiliations:** 1 Department of Prosthodontics, Stomatological Hospital, Tianjin Medical University, Tianjin, China; 2 School of Materials Science and Engineering, Tianjin University, Tianjin, China; Meridional Faculty IMED, BRAZIL

## Abstract

All-ceramic restoration has become a popular technology for dental restoration; however, the relative bond strength between the ceramic and resin limits its further application. Long-term high bond strength, especially after thermal cycling, is of great importance for effective restoration. The effect of physical and/or chemical surface treatments on bonding durability is seldom reported. To overcome this problem, we investigate the bond strength between lithium disilicate ceramics (LDC) and two kinds of resin cements before and after thermal cycling for a variety of surface treatments including hydrofluoric acid, two kinds of silane and a combined effect. The shear bond strength in every group is characterized by universal mechanical testing machine averaged by sixteen-time measurements. The results show that when treated with HF and a mixed silane, the LDC surface shows maximum bonding strengths of 27.1 MPa and 23.3 MPa with two different resin cements after 5000 thermal cycling, respectively, indicating an excellent ability to resist the damage induced by cyclic expansion and contraction. This long-term high bond strength is attributed to the combined effect of micromechanical interlocking (physical bonding) and the formation of Si-O-Si and -C-C- at the interface (chemical bonding). This result offers great potential for enhancing bond strength for all-ceramic restoration by optimizing the surface treatment.

## Introduction

All-ceramic restoration has become a popular technology to meet the increasing requirements of dental restoration [1,2]. Compared with traditional metal-porcelain, all-ceramic restoration exhibits a series of outstanding properties, including biocompatibility, low thermal conductivity, high translucency, good chemical stability and similar coefficients of thermal expansion to that of teeth [3]. Compared with zirconia (CAD/CAM) systems, hot-pressed lithium disilicate ceramics (LDC) show great potential for ceramic monolithic restoration. Hot-pressed LDC consisting of a silica glass matrix and lithium oxide (Li_2_O) not only provides better translucency and aesthetics than zirconia ceramic but also has better flexural strength than leucite-reinforced glass ceramics [4,5].

According to previous studies, clinical restoration mainly depends on the bonding effect between the ceramic and resin cement rather than the strength of the ceramic [6,7]. Strong interfacial bonding between the ceramic and resin cement increases the fracture resistance [8] and marginal adaptation [9,10] and reduces the microleakage [10,11], resulting in the retention of the restoration. Surface modification to increase the roughness or to form specific chemical bonds is of great importance for increasing interfacial bonding. However, due to the relatively low strength, LDC usually suffers from serious surface damage introduced by traditional sandblasting abrasion, leading to a decrease in flexural strength [12,13]. Hydrofluoric acid (HF) treatment is considered as a relatively mild method to chemically modify LDC [14, 15]. HF can etch the surface to create an irregular microstructure on the surface [16], resulting in a high specific surface area that increases the bonding area at the interface [17–22]. Furthermore, silane coupling is another effective way to increase the bonding effect by forming siloxane bonds at the interface between the ceramic and resins. A silane solution of organofunctional trialkoxysilane esters can copolymerize with the remaining C = C bonds of the resin cement. Importantly, hydrolyzed alkoxy groups of silane can react with hydroxyl groups of the LDC surface to form covalent siloxane bonds. In general, 3-methacryloxypropyletrimethoxysilane (3-MPS) is used as a silane coupling agent to improve the wettability and free energy of the ceramic surface [23]. Our previous studies reported that HCl favored the hydrolyzation of 3-MPS and that a mixed silane of 1,2-Bis(trimethoxysilyl) ethane (BTE) and 3-MPS could significantly enhance the adhesion between the resin and ceramic [23–26]. This universal adhesive resin cement (Panavia F 2.0) contains a phosphate ester monomer, 10-methacryloxydecyl dihydrogen phosphate (MDP), which is effective in establishing a relatively stable chemical bonding with the ceramic. Thus, this resin cement offers great potential to improve bond strength at the interface between the ceramic and dentin [27,28]. Importantly, both physical interlocking and chemical bonding can decrease along with cyclic expansion and contraction at high and low temperatures. This effect with the water microleakage induced by chemical degradation at the interface might result in the separation of resin cement from the ceramic [29]. Thus, this bond strength after thermal cycling (TC) influences the long-term restoration. Despite initial progress, the bond durability between LDC and resin and systems controlled by different treatments has been seldom reported.

Herein, we tried to illustrate the effect of physical and/or chemical surface treatments on bonding durability. The bond strength between LDC and two kinds of resin cements before and after thermal cycling upon a variety of surface treatments including HF, two kinds of silanes (commercial or experimental ones) or a combined treatment was investigated. The shear bond strength in every group is characterized by universal mechanical testing machine. Morphologies of the bonding interface and fracture section were observed by SEM and optical microscopy.

## Materials and methods

### Preparation of specimen

Lithium disilicate ceramic blocks (IPS e.max Press, Ivoclar Vivadent, Schaan, Liechtenstein) were fabricated by using the lost-wax and hot-pressing technique according to the manufacturer’s instructions. The blocks (100× 100 × 20 mm) were cross-sectioned into about 400 ceramic plates (5× 5 × 2 mm) under water-cooling at a speed of 800 rpm and a fixed load of 200 g (Isomet 1000, Buehler, Illinois, USA). Then, the plates were embedded in self-curing pour resin (Shofu, Tokyo, Japan). The plate surfaces, which were used as adherents, were polished using a sequence of 600- and 1,000-grit silicon carbide papers under running water. The specimens were then cleaned with 10% citric acid in an ultrasonic bath for 20 min in order to remove loose impurities. Materials brands, abbreviations, and the manufacturers of the materials are shown in Table 1.

**Table 1:** Materials brands, abbreviations, manufacturers of the materials used in this paper.

Material	Composition	Manufacture	Lot number /Code
**IPS e.max Press**	SiO_2_,Li_2_O,K_2_O,MgO,ZnO_2_, Al_2_O, P_2_O_5_	Ivoclar Vivadent, Liechtenstein	N/A /LDC
**Silane solution 1**	3-methacryloxypropyle- trimethoxy silane	ShinEtsuChemical, Industry,Tokyo, Japan	901770 /3-MPS
**Silane solution 2**	1,2-Bis(trimethoxysilyl)- Ethane	Tokyo Chemical Industry, Tokyo, Japan	HH3SE /BTE
**Hydrofluoric acid**	4.5% hydrofluoric acid	Ivoclar Vivadent, Liechtenstein	V12791 /HF
**Monobond-s**	Ethanol:50–100%,3-methacryloxypropyle-rimethoxy silane<2.5%	Ivoclar Vivadent, Liechtenstein	S05674
**Panavia F 2.0**	MDP, HHD, BP, CQ, etc	Kuraray, Tokyo, Japan	071200 / N/A
**PermaCem-Dual**	Bis-GMA, MBP, silica, etc.	DMG, Hamburg, Germany	790199 /N/A

MDP: 10-methacryloyloxydecyl hydrogen phosphate, HHD: hydrophobic and hydrophilic dimethacrylate, BP: benzoyl peroxide, CQ: camphoroquinone, Bis-GMA: Bisphenol A Dyglicidil Metacrilate, MBP: 2-hydroxy-4-methoxybenzophenone

### Preparation of experimental primer

As per the previous study [23,26], the experimental silane agent consisted of primer A and primer B. Primer A was prepared by dissolving a silane mixture (50 mg) in ethanol (1 ml). The additional amount of BTE (Tokyo Chemical Industry, Tokyo, Japan) added to 3-MPS (ShinEtsu Chemical Industry, Tokyo, Japan) was 30 mol%. Primer B was prepared by using hydrochloric acid solution. Hydrochloric acid solution (0.1 mol/L, Wako Pure Chemical Industries, Osaka, Japan) was diluted with distilled and deionized water (pH = 6.0) until the pH value of the hydrochloric acid solution was 1.70. After that, the diluted hydrochloric acid solution was mixed in ethanol by 50 vol%, which was obtained as the experimental primer B.

### Preparation of adherent specimens

**Fig 1:**
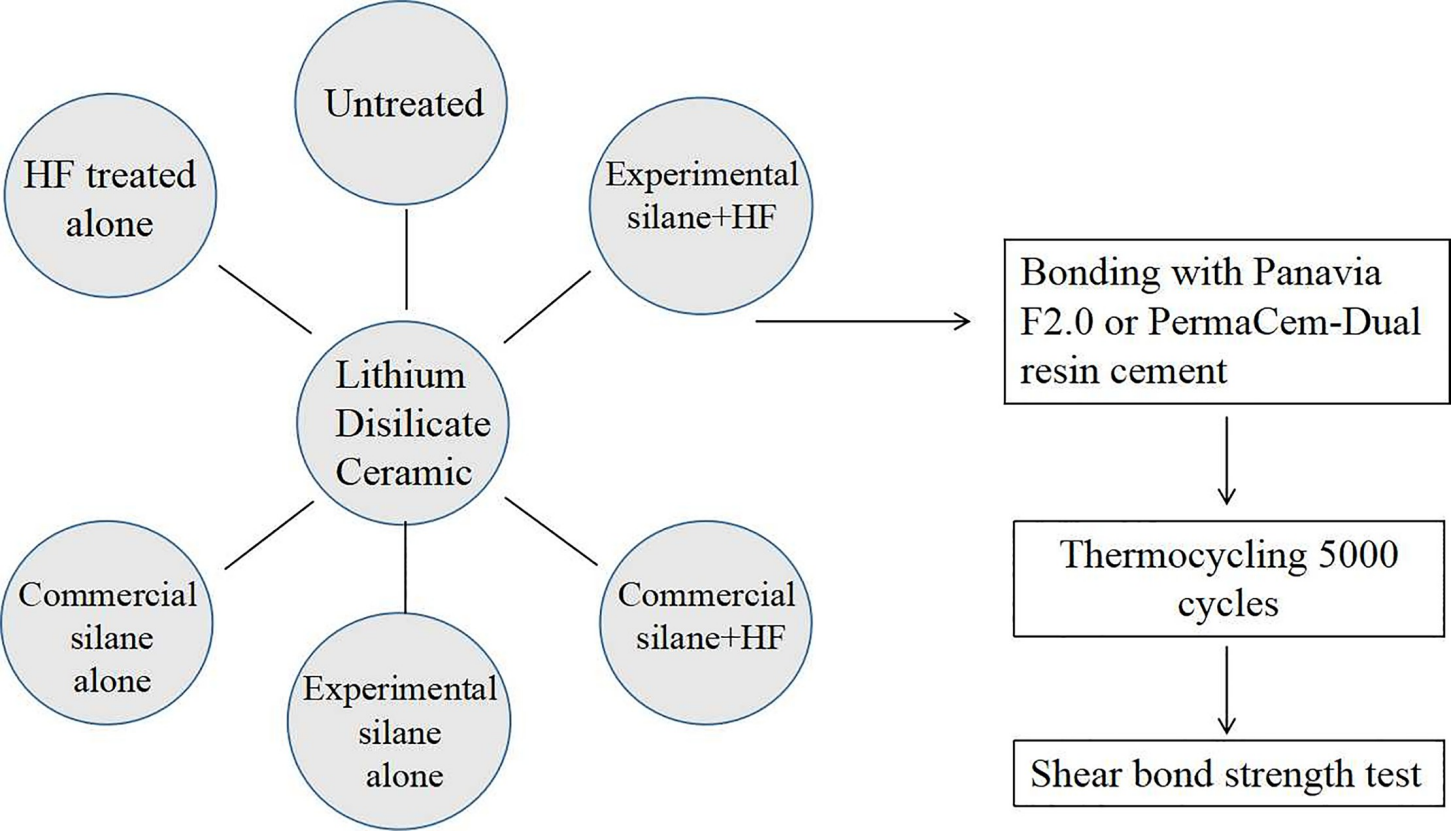
The schematic test protocol.

According to different surface treatments and resin cements, 12 groups were designed and studied in total, as shown in Fig 1. The bond strength between LDC and two kinds of resin cements (Panavia F 2.0 and PermaCem-Dual) was measured, analyzed and discussed to investigate the effect of the HF, two kinds of silanes (commercial or experimental ones) or a combined treatment on it. Each group including 32 samples was divided into two subgroups, before and after thermal cycling. The shear bond strength in each subgroup was characterized by universal mechanical testing machine averaged by sixteen-time measurements. The details were shown below.

Group 1 (UTP): No treatment was applied onto the surface of LDC. A silicone ring mold was placed onto the LDC surface. The silicon ring is a circular hole with an inner diameter of 3 mm and a thickness of 2 mm. Then, the inside of the silicon ring’s circular hole was filled with a dual-curing resin cement Panavia F 2.0 (Kuraray, Tokyo, Japan). Then, the specimen was irradiated with visible light (Coltolux LED, Coltene, OH, USA) for 30 s. The power density of the light source was 300–500 mW/cm2, which was determined using a 3M Light Checker (3M Health Care, Tokyo, Japan). After irradiation, the residual resin cement and the ring mold was carefully removed.

Group 2 (UTPC): No treatment was applied onto the surface of LDC. Adhesion of resin cement was also carried out according to the description of the previous experimental group 1, except that the type of adhesive resin as changed to PermaCem-Dual (DMG, Hamburg, Germany) resin cement.

Group 3 (HP): The LDC surface was pretreated with HF (Ivoclar Vivadent, Schaan, Liechtenstein) for 20 s. Followed by rinsing with distilled water for 1 min, and ultrasonically cleaned for 10 min. After dried with compressed air, the treated surface was bonded by using the Panavia F 2.0, as described above.

Group 4 (HPC): The LDC surface was subjected to the same and bonding procedure as in group 3, with PermaCem-Dual resin cement used for bonding to the LDC surface.

Group 5 (CP): One bottle type of a commercial silane coupling agent (Monobond-s, Ivoclar Vivadent, Liechtenstein) was applied onto the LDC surface for 60 s, and then the surface was dried in air for 10 s with a gentle air stream. In this group, Panavia F 2.0 was used as the adhesive resin cement.

Group 6 (CPC): PermaCem-Dual was used as the adhesive resin cement in this group, and the pretreatment procedure for the LDC surface was the same as that used for group 5.

Group 7 (EP): For this group, a two-bottle type experimental silane agent was used for the silanization of the LDC surface. The same amount of Primer A and Primer B was mixed in a mixing dish for 10 s. Thereafter, the mixed silane agent (0.3 mg) was applied onto the LDC surface for 1 min. This 1 min is the period for the evaporation of ethanol at room temperature (23 ± 1°C). Then, the silanated LDC was dried at room temperature for 2 min. As in the bonding procedure described above, Panavia F 2.0 was used as the adhesive resin cement in this group.

Group 8 (EPC): PermaCem-Dual was used as the adhesive resin cement in this group, and the pretreatment procedure for the LDC surface was the same as that used for group 7.

Group 9 (HCP): For this group, the LDC surface was pretreated with HF. After HF was air dried, commercial silane (Monobond-s) was applied onto the surface as the silanization procedure. Panavia F 2.0 was used as the adhesive resin cement in this group.

Group 10 (HCPC): PermaCem-Dual was used as the adhesive resin cement in this group, and the pretreatment procedure for the LDC surface was the same as that used for group 9.

Group 11 (HEP): For this group, the LDC surface was pretreated with HF. After the HF was air dried, experimental silane was applied onto the surface as the silanization procedure. Panavia F 2.0 was used as the adhesive resin cement in this group.

Group 12 (HEPC): PermaCem-Dual was used as the adhesive resin cement in this group, the pretreatment procedure for the LDC surface was the same as that used for group 12.

### Measurement of the shear bond strengths before and after thermo-cycling

After the irradiation of the resin cement bonded onto the LDC surface, all the bonded samples were immersed in water at 37°C for 1 day. Each group including 32 samples was divided into two subgroups (n = 16): before and after thermo-cycling. The samples were thermocycled between 5°C and 55°C in a water bath at 5,000 cycles for each bath (TC-501F, Weier, Suzhou, China). The dwell time in the water bath was 60 s. The transfer period was 7 s.

**Fig 2:**
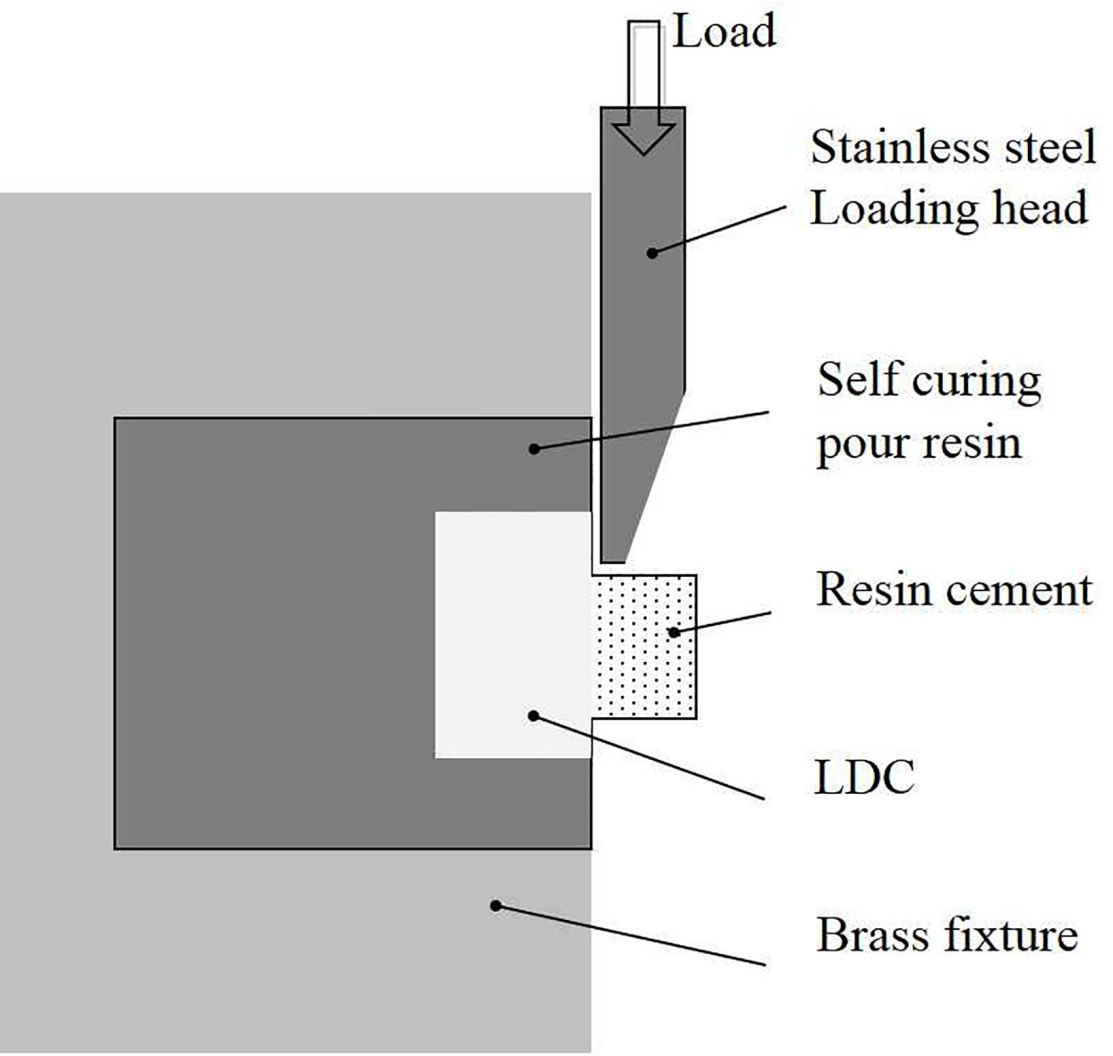
Schematic illustration of the shear bond strength test.

The shear bond strength (SBS) of the resin for the LDC surface was measured under a crosshead speed of 0.5 mm/min by a universal testing machine (Instron 3367, MA, USA, Fig 2). The SBS were calculated in units of MPa by dividing the load at failure by the surface area (mm^2^) of each sample. The maximum load recorded in the shear bond test indicates the degree of retention. If premature failure occurred before bond strength testing, the bond strength was believed to be 0 MPa. A cross-sectional view of the shear bond strength test is illustrated in Fig 2. The number of bonded specimens in each group was 16.

### SEM observation

**Fig 3:**
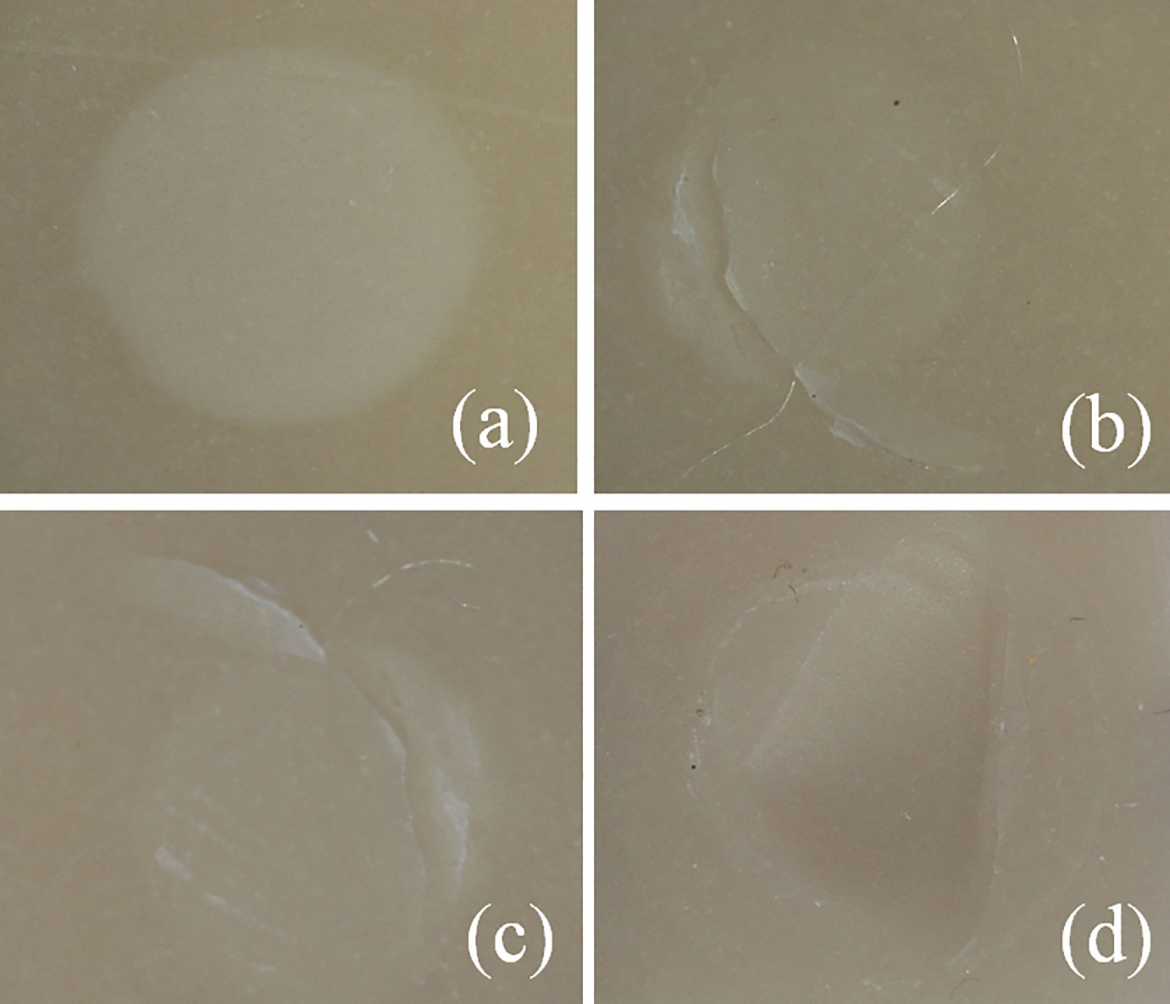
Optical image of the fracture surface indicating the mode of (a) interfacial failure, (b, c) mixed failure and (d) cohesive failure.

The surface morphology of the LDC sample before and after HF etching and their bonding performance between the resin cement was observed by SEM (Hitachi S-4800, Fig 3) using the backscatter detector. The surface is highly polished before the observation. All of the samples were observed after being coated with gold.

### Determination of the failure mode

**Fig 4:**
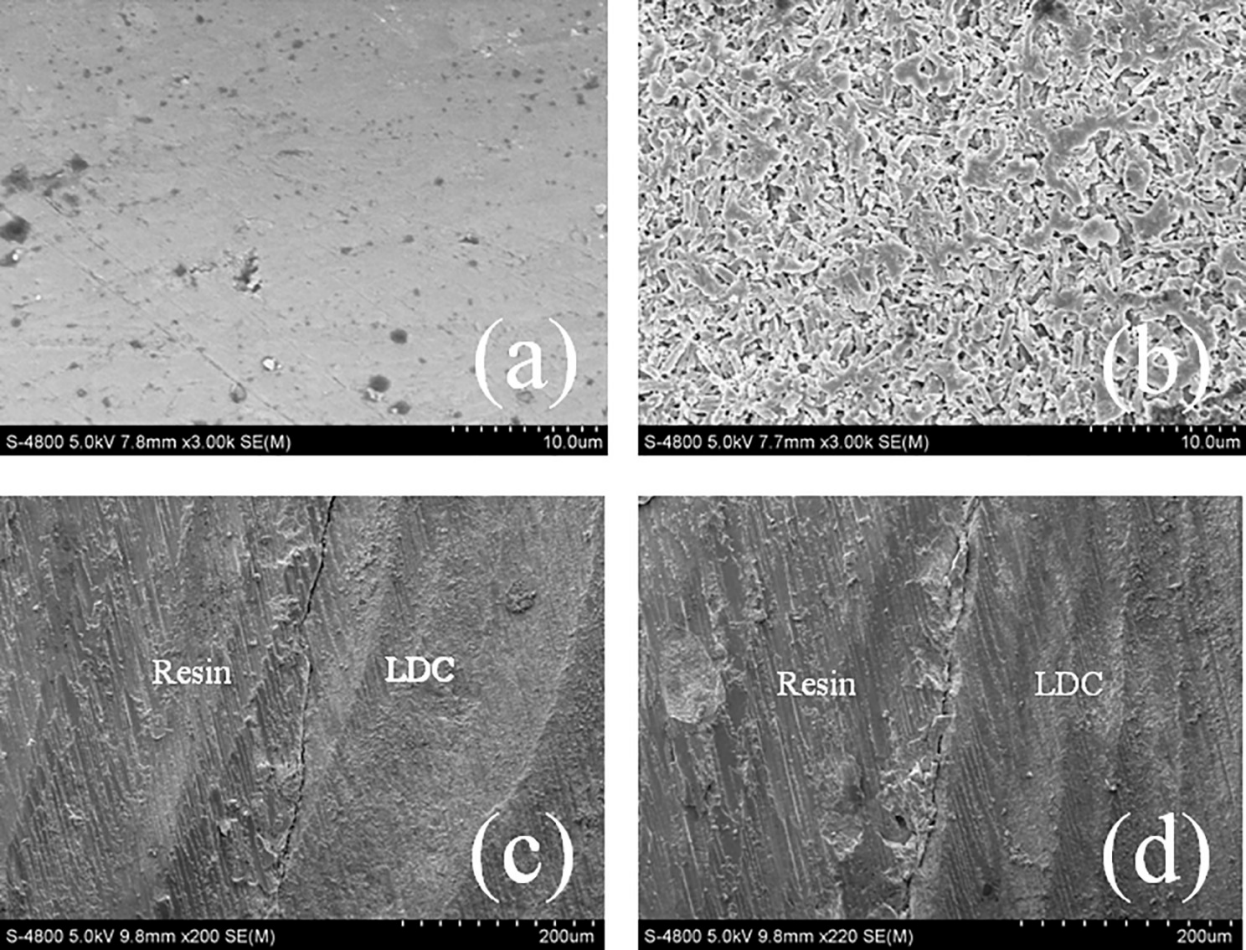
SEM images of surface morphology for LDC (a) before and (b) after HF treatment, and the bonding interface between the resin cement and LDC surface after TC (c) without and (d) with the HF treatment.

After SBS testing, a digital microscope (MSV 330, Anyty, 3R, Japan) was used to observe the fracture. The failure mode for all specimens is illustrated in Fig 4. The modes were classified into three fracture types: 1) cohesive failure of the LDC, as shown in picture d. 2) mixed failure consisting of interfacial failure and cohesive failure, as shown in picture b and c. 3) interfacial failure at the interface between the LDC and resin cement, as shown in picture a.

### Statistical analysis

The average shear bond strength and its standard deviation (SD) were calculated for each group, with and without thermocycling. Statistical analysis consisted of one-way analysis of variance (ANOVA) in conjunction with Tukey’s post hoc test for multiple comparisons (SPSS Software, San Diego. CA, USA) with a significance level of p < 0.05. The significance of differences between groups was determined by a Student’s test for each independent variable, and ANOVA tests were performed with all samples [30–32]

The fracture mode for all experimental groups was analyzed by the complex chi-square (χ^2^) test to determine significant differences in the three types of fracture modes (the typical image is shown in Fig 4). Statistical significance was set at the 0.05 level.

## Results and discussion

The bond strength between the LDC and the two kinds of resin cements controlled by the treatment of HF and silane (experimental or commercial), respectively, is shown in Table 2. The two resin cements exhibit an identical changing trend for the bond strength after the treatment. Before TC, the lowest bond strength of 4.3 MPa and 3.5 MPa was obtained in the group for UTP and UTPC, respectively. This result indicates that the untreated LDC surface exhibits weak bonding with the resin cement. HF treatment results in a more than two-fold increase (p<0.05) in the bond strength (14.7 MPa for Panavia F cement and 10.9 MPa for PermaCem-Dual cement) compared with the untreated surface. This treatment is more effective for improving the bond strength than that treatment using commercial silane (12.1 MPa for Panavia F cement and 7.4 MPa for PermaCem-Dual cement). Moreover, the treatment with experimental silane (16.1 MPa for Panavia F and 14.7 MPa for PermaCem-Dual) leads to a much higher bond strength than the treatment with HF due to the formation of chemical bonds (-Si-O-Si- and -C-C-) at the interface. This effect can be further improved by dual treatment. Finally, for treatment with both HF and experimental silane, the LDC surface shows the highest bond strength of up to 33.4 MPa and 28.7 MPa with Panavia F cement and PermaCem-Dual cement, respectively, which is a 676.7% and 720.0% increase, respectively, compared to the untreated surface (significance difference). This result indicates that controlling the treatment effectively increases the bond strength between LDC and resin cement.

**Table 2:** Shear bond strength between the lithium disilicate ceramic surface and resin cement before and after thermo-cycling.

Resin Cement	Group	Bond Strength (MPa)		Type of fracture mode [1/2/3]	
		Before TC	After TC	Before TC	After TC
**Panavia F 2.0**	1 (UTP)	4.3(1.2)^1^_a_	0.2(0.1)^2^_a_	[0/0/16]^1^_a_	[0/0/16]^1^_a_
	3 (HP)	14.7(2.9)^1^_c_	2.1(1.2)^1^_b_	[7/6/3]^1^_b_	[0/0/16]^2^_a_
	5 (CP)	12.1(3.7)^1^_c_	4.3(2.0)^2^_b_	[8/5/3]^1^_b_	[0/1/15]^2^_a_
	7 (EP)	16.1(3.5)^1^_c_	7.5(2.1)^2^_c_	[10/5/1]^1^_b_	[0/4/12]^2^_a_
	9 (HCP)	25.2(2.8)^1^_d_	15.1(3.6)^2^_d_	[16/0/0]^1^_c_	[7/6/3]^2^_b_
	11 (HEP)	33.4(4.1)^1^_f_	27.1(3.3)^2^_e_	[16/0/0]^1^_c_	[16/0/0]^1^_c_
**PermaCem-Dual**	2 (UTP)	3.5(0.7)^1^_a_	0.0(0.0)^2^_a_	[0/0/16]^1^_a_	[0/0/16]^1^_a_
	4 (HP)	10.9(2.1)^1^_c_	2.3(0.8)^2^	[6/5/5]^1^_b_	[0/0/16]^2^_a_
	6 (CP)	7.4(1.5)^1^_b_	3.7(1.1)^2^_b_	[2/4/10]^1^_a_	[0/0/16]^2^_a_
	8 (EP)	14.7(2.8)^1^_c_	8.2(1.9)^2^_c_	[9/4/3]^1^_b_	[0/1/15]^2^_a_
	10 (HCP)	23.9(3.5)^1^_d_	14.5(2.8)^2^_d_	[16/0/0]^1^_c_	[8/6/2]^2^_b_
	12 (HEP)	28.7(3.4)^1^_e_	23.3(2.8)^2^_e_	[16/0/0]^1^_c_	[15/1/0]^1^_c_

(): SD. For each horizontal row in the value of the bond strength: superscript values with different numbers indicate a statistically significant difference (P<0.05). For each vertical column in the mean value of the bond strength: subscript characters with same letters (a-f) indicate no statistically significant difference (P>0.05). [1/2/3]: 1) cohesive failure, 2) mixed failure consisting of interfacial failure and cohesive failure, and 3) interfacial failure. The sample size for each experimental group was 16.

The bonding effect between the ceramic surface and resin cement is also demonstrated by the change in fracture mode after different treatments. The type of fracture mode changed from an all-interfacial failure (Fig 4A) for the untreated surface (groups 1 and 2) to a mixed (Fig 4B and 4C) or cohesive (Fig 4D) failure of some specimens when the LDC surface was treated with HF (group 3 and group 4) or with commercial and experimental silane alone (group 5, 6, 7 and 8). Furthermore, when treated with both HF and silane, all specimens showed cohesive failure. This result is consistent with the change trend for the increasing bond strength.

High strength ensures firm bonding between LCD and resin; however, the change in the surface bonding during thermal cycling determines the effect of long-term ceramic restoration. Previous studies have focused on the bond strength between ceramics and resin cement [23–25], but there are few reports on the bond durability for surface damage induced by expansion or contraction. In this paper, we investigated the bond durability influenced by different treatments (HF, silane or a combined effect) for two resin cements.

The bond strength between the LDC and resin after 5000 TC was investigated (Table 2). It can be seen that all LDC surfaces showed a significant decrease (p<0.05) [*R = (bonding strength before TC—bonding strength after TC) / bonding strength before TC*] in the bond strength with the resin after TC regardless of the treatment. For the untreated groups 1 and 2 (UTP and UTPC), the resin was separated from the LDC surface with no bond strength. When treated with HF, the bond strength seriously decreased to below 3.0 MPa with an ***R*** of up to 85.5% (14.7 → 2.1 MPa in group 3) and 78.9% (10.9 MPa → 2.3 MPa in group 4) after TC. Further, the surface treatment with both commercial and experimental silane also showed an obviously reduced bond strength (4.3 and 7.5 MPa for Panavia F cement, 3.7 and 8.2 MPa for PermaCem-Dual), although these values are higher than that obtained after HF treatment (group 3 and 4). Interestingly, the surface treated with experimental silane exhibits a relatively low decrease in bond strength (***R*** = 53.5% in group 7 and 44.5% for group 8) than that obtained for treatment using the commercial silane (***R*** = 64.5% in group 7 and 50% for group 8).

When treated with HF and experimental silane, the maximum bond strength between the LDC and resin cement reaches up to 27.1 MPa and 23.3 MPa after TC, which is approximately 18–20% lower than that before TC. The high bond strength is also 1190.4% (913.0%) and 261.3% (184.1%) higher than that obtained for the HF- (2.1 MPa, 7.5 MPa) and experimental-silane (2.3 MPa, 8.2 MPa) treated surface for Panavia F cement (PermaCem-Dual cement). This result shows that the combined effect of micromechanical interlocking (physical bonding) and the formation of Si-O-Si and -C-C- at the interface (chemical bonding) is more effective to resist the damage induced by expansion or contraction during thermal cycling. We also found that, after all treatments, the bond strength between the resin and LDC surface showed no significant difference before and after TC except for the HF and silane treatments (group 11 and group 12) before TC.

The increase in bond strength between the LDC and resin cement is mainly attributed to the enhanced physical or chemical interaction at the interface. The bonding durability was determined by the ability to resist the damage induced by expansion or contraction. Specifically, HF could dissolve the glassy matrix of the ceramic to create an irregular microstructure on the LDC surface. This effect increased the surface area for micromechanical interlocking and bonding, resulting in an increased bond strength. However, after TC, the specimens showed poor bonding durability with interfacial failure. This result indicates that such micromechanical interlocking on high-area cohesive areas is not strong enough to resist thermal fatigue because the difference in the thermal expansion coefficient between the resin and LDC caused separation at the interface.

Despite a relatively low bond strength (group 5 and 6) before TC, the bond strength could also be enhanced by treating the LDC surface using both experimental and commercial silane after TC. This result indicates that the formation of chemical bonds helps to resist the damage induced by expansion or contraction during TC. Furthermore, the surface treated with experimental silane also exhibited a higher bond strength than that treated both before and after TC.

The experimental silane used in this study was a two-bottle type. HCl was utilized to accelerate the hydrolysis of the methoxy portion in the silicone-functional group in the mixed silane groups (23). The mixed silane consisting of 3-MPT and BTE was used for surface treatment. The 3-MPT is a chemical bridge between the LCD and resin cement. The -C = C- of 3-MPT polymerizes with the resin for chemical -C-C- bonding. Furthermore, the LDC can react with the silane to form -Si-O-Si- chemical bonds, leading to the formation of an organic layer anchored on the LCD surface. As a result, the cross-linking at the interface increases the bond strength between the LCD and resin cement (24–25). Thus, the nature of the organic layer determines the bond strength. The cross-linking is further enhanced by using the two-bottle experimental primer. The BTE not only can form a large amount of -Si-O-Si- chemical bonds with the LDC but also generates an elastic organic layer at the interface between the LDC and resin cement. This elastic layer is useful to resist expansion and contraction during TC (26). In addition, it is well known that the silane agent is an excellent wetting agent, which effectively increases the bond strength because it improves the intimacy of the surface contact with the cement. As a result, both HF and experimental silane treatment resulted in the maximum bond strength between the LDC and resin cement due to micromechanical interlocking and the formation of Si-O-Si at the interface.

This analysis is also confirmed by the fracture mode. The fracture mode of the specimen treated with HF and silane alone changed significantly from cohesive or mixed failure to interfacial failure. In contrast, the fracture mode after dual treatment (group 11 and 12) changed from all cohesive failure to mainly cohesive or mixed failure (significant difference). This result is consistent with high bond strength after TC. However, it is noteworthy that the fracture mode is still almost cohesive, and there are significant differences with all other groups.

Fig 4 shows the surface morphologies of the LDC surface (a) before and (b) after HF treatment. It can be seen that the treatment generated a large number of voids, which are attributed to the dissolution of the glass phase of the ceramics. This effect resulted in the formation of a microporous irregular structure on the surface of the ceramics, which increased the bonding surface area between the ceramics and resin cement and promoted micro retentions on the ceramic surface, which was ideal for micromechanical interlocking of the resin cement.

SEM images of the adhesive interfaces between the LDC surface and resin cement before and after TC were also observed. It can be seen that there were obvious cracks in the bonding interface between the LDC ceramics and resins after TC whether or not the LDC ceramics were etched by HF. HF treatment resulted in a rough surface (Fig 4D) with uneven cracks compared to the treatment without HF (Fig 4C) after the fatigue test. This irregular microstructure favors micromechanical interlocking between the resin and LDC, which favors strong bonding adhesion at the interface.

## Conclusion

We investigated the effect of a variety of physical and chemical surface treatments on the bond strength between LDC and resin cements before and after TC. After HF treatment, the LDC surface showed a large number of voids and cracks, with a high specific surface area for both interlocking and chemical bonding. All the treatments, including HF, silane and a combined treatment increased the bonding strength; however, this effect seriously decreased after TC. This result reveals that chemical bonding is more effective in resisting thermal damage than physical interaction. As a result, we obtained a maximum bond strength of 27.1 MPa and 23.3 MPa after TC for Panavia F and PermaCem-Dual cement, respectively. The long-term bonding durability for resisting the damage induced by cyclic expansion and contraction is attributed to the combined effect of micromechanical interlocking (physical bonding) and the formation of Si-O-Si and -C-C- at the interface (chemical bonding). This result offers great potential for enhancing the bond strength for all-ceramic restoration by optimizing the surface treatment.
